# Heavy metals and radon content in spring water of Kosovo

**DOI:** 10.1038/s41598-020-67371-1

**Published:** 2020-06-25

**Authors:** Smiljana Marković, Biljana Vučković, Ljiljana Nikolić-Bujanović, Sanja Mrazovac Kurilić, Nataša Todorović, Jovana Nikolov, Anja Jokić, Boban Đokić

**Affiliations:** 1 University of Priština-Kosovska Mitrovica, Faculty of Technical Sciences, Knjaza Miloša No. 7, Kosovska Mitrovica, Serbia; 2 University of Priština-Kosovska Mitrovica, Faculty of Sciences, Lole Ribara No. 29, Kosovska Mitrovica, Serbia; 3 IHIS Techno-Experts, Batajnicki put 23, 11080 Belgrade, Serbia; 4 University “Union-Nikola Tesla”, Faculty of Ecology and Environmental Protection, Belgrade, Serbia; 5 Department of Physics, University of Novi Sad, Faculty of Sciences, Trg Dositeja Obradovića 4, Novi Sad, Serbia

**Keywords:** Ecology, Environmental sciences, Hydrology, Natural hazards, Health care, Risk factors, Chemistry

## Abstract

Paper presents results of researches carried out on various locations and immediate vicinity of mining and industrial activities of the northern and south-eastern part of Kosovo. Concentrations of As, Cd, Cr, Cu, Fe, Mn, Ni, Pb, Zn, Rn-222, as well as temperature and pH values of natural spring water were measured at 15 measuring sites (that belong to Zvečan, Leposavić and Novo Brdo municipalities), in April–May and September–October 2019. The quantification of heavy metals’ content was performed by applying ICP-OES method. In analysed samples a high content of As, Pb, Fe and Ni was found. Carcinogenic and non-carcinogenic risks due to the content of heavy metals in water were evaluated. Concentration of radon in water was measured by the alpha spectrometric method, and measured values range in the interval from 0.34 ± 0.12 to 341 ± 35 Bq/L. The yearly doses of inhalation and ingestion were determined for the measured concentrations of radon. Mutual correlation by the Pearson correlation coefficient, principal component analysis, cluster analysis and spatial distribution analysis of the researched parameters of sampled water were done. The most expressed mutual dependence of some heavy metals leads to the conclusion that they have the same anthropogenic origin.

## Introduction

There are many spring water in the areas of industrial and mining activities, worldwide and in Kosovo, too. Population can use them for drinking, or for some other purposes. The cause of the water pollution near the mentioned areas are the tailing ponds of mines which have a heterogenic chemical composition due to the content of heavy metals, especially As, Cd, Cr, Cu, Fe, Mn, Ni, Pb and Zn. Their influence on surface water and groundwater is shown through the complex processes of dissolution, infiltration, mobilization and biogeochemical cycling^1,2^. Heavy metals are not the subject of the process of self cleansing, because their concentration during migration is only diluted^3–5^. Heavy metals in water can be found as ions in the form of easily diluted compounds. Taken into the organism, they adhere to enzymes, inhibiting their function, where they can cause numerous physiological and neurological consequences^6,7^. Temperature and pH value affect the concentration of heavy metals, so at lower pH values (4–7) and higher temperatures the release of metals from mineral grains is more intense^8^.

Numerous epidemiological studies have shown that the presence of radon in the surroundings is considered the second most important cause of the lung malignity (right behind the long-term exposure to tobacco smoke)^9–12^. It can be taken into organism in two ways: by inhalation and ingestion. The health risk of radon in drinking water is usually low compared to the total inhaled radon, but since radon is an aquafobic, by exhalation from water increases its presence in closed facilities, and at the same time increases its detrimental influence on health. Radon is usually present in significant concentrations in those groundwater that have been in contact with granite rocks, slates, as well as sandstone and limestone. Other factors also influence the concentration of radon, such as the following: the paths of water circulation, the presence of soluble gasses, temperature and pressure^13^. Temperature is a parameter that, by its value, can be an indicator of the increased presence of radon—increasing temperature decreases the solubility of radon in water^14^. The value of pH is a parameter that can be indirectly affected by the presence of radon in the water, because radium more easily and more quickly dissolves in water with a lower pH value. Therefore, the effect of water pH on radon concentration was also monitored in this study^15^. When the water rich with radon is used for drinking, or some other purposes, directly from the spring, there is a potential risk for population’s health. Thus it is very important to research and control the concentration of radon in natural spring water and determine if they are radiological safe.

The aim of this paper is to determine the presence and to find out the content of heavy metals and radon in samples of natural spring water at 15 measuring sites from rural areas, at three municipalities: Leposavić, Zvečan (northern part of Kosovo) and Novo Brdo (south-eastern part of Kosovo), as well as to determine their potential risk for people’s health in the researched areas. In studied rural areas, population is supplied with water from existing natural sources. Water is used in daily life for drinking and other purposes. Results of these researches give the first picture of the spring water quality and can serve as foundation for formation a map of health wholesomeness of water from the territory of Kosovo.

## Study area

**Figure 1:**
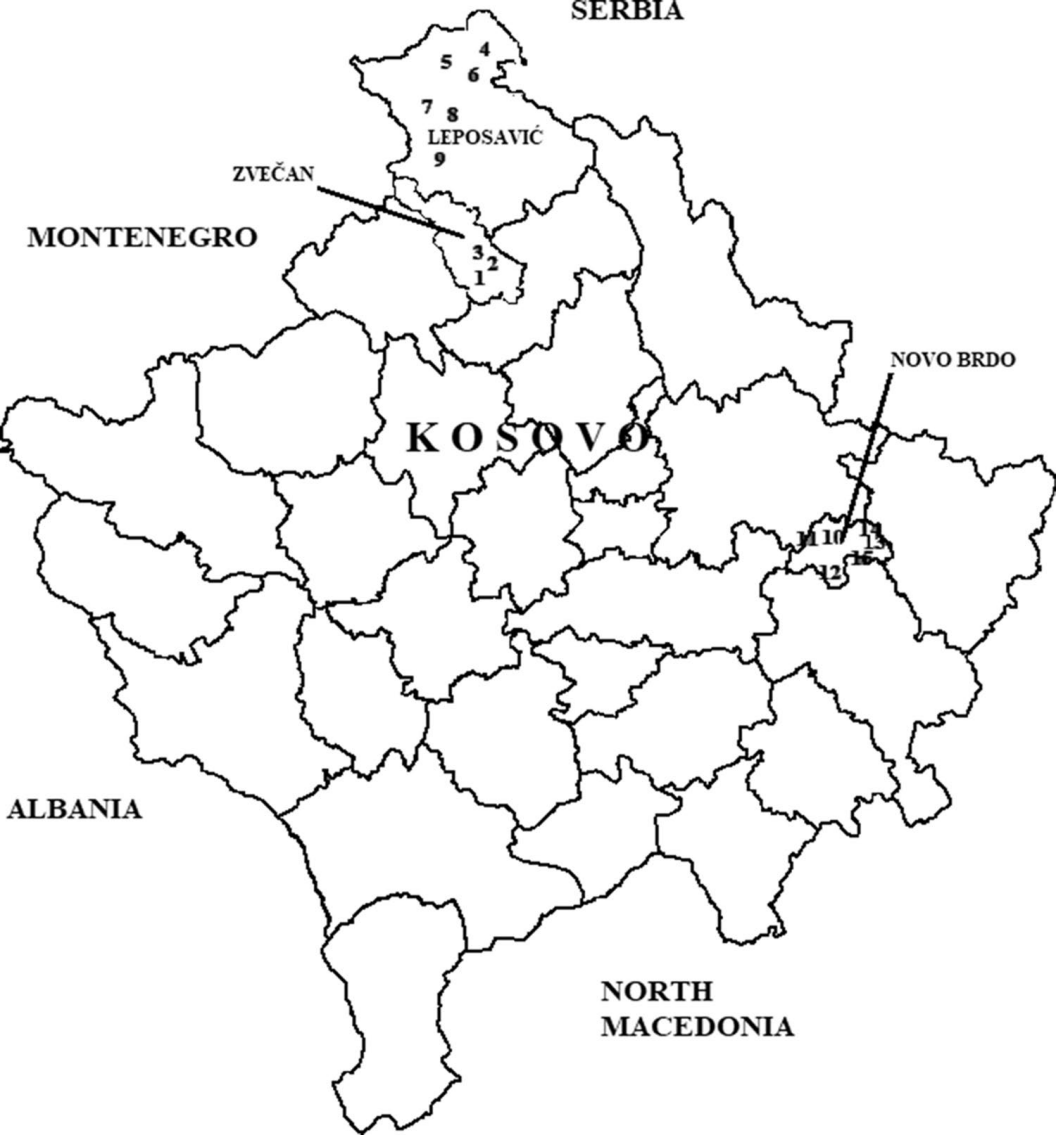
Areas of investigated natural spring waters (QGIS 3.12.2 https://qgis.org/en/site/forusers/download.html).

Map of 15 measuring sites which are situated in the surroundings of mining and industrial activities, is shown in the Fig. 1. Three locations are situated near the MMCC (mining-metallurgical and chemical combine) “Trepča” (the municipality of Zvečan), six of them are situated in the vicinity of mines which are rich in lead and zinc ores: Crnac, Žuta Prla, Koporiće, Belo Brdo (the municipality of Leposavić) in the northern part of Kosovo. Six researched locations are situated in the mining area of the municipality Novo Brdo, in the south-eastern part of Kosovo. Central place to this municipality is the Novo Brdo mine, which is rich in lead, zinc, silver and gold minerals^16,17^.

### Geological background

Geology of the study area is heterogeneous comprising a diverse lithostratigraphic units that formed within the period between the Early Paleozoic and Tertiary. Regarding geotectonic setting, the study area is a part of the Western Vardar zone (VZ), whereas some localities included in this work, are placed at the immediate border of the VZ with the Kopaonik block ridge and the Drina-Ivanjica unit, a tectonic element of Dinarids^18,19^. Vardar zone represents the Mesozoic suture zone in the central part of the Balkan peninsula and is built by Jurassic ultrabasic rocks, ophiolite mélange and metamorphic rocks of different grade overlain by the Upper Cretaceous-Paleogene flysch^19^. The youngest products are Neogene and Quaternary sediments and Cenozoic magmatic rocks. The former deposited in the Kosovo basin and in valleys of long rivers, such as the River Ibar. Cenozoic magmatic suite is associated with numerous Pb–Zn–Ag deposits and occurrences, as in the case of the Belo Brdo ore field. Metamorphic rocks in Belo Brdo include: gneisses, amphibolites, quartz-mica and amphibolite schists, and marbles^20^.

The recognized tectonic structures are synclines, anticlines and trench-synclines, among the folded ones, and thrust sheets and faults. Regional geology had the influence to spatial distribution of groundwater. High diversity of lithostratigraphic units in the area of interest, as has been mentioned, led to variously distributed aquifers depending on the rock porosity. Though, fissure aquifers developed in metamorphic and volcanic rocks (serpentinites, quartzlatites, basalts, etc.). Rocks on/or near surface subjected to physical–mechanical weathering allowed water to accumulate in variable systems of fissures and cavities, depending on the environmental conditions and rock permeability. A part of the examined natural spring waters at measuring stations within the municipality Leposavić and Zvečan are placed in ultramafic rocks of southwestern slope of the large Ibar peridotite massif (serpentinized harzburgites prevail, less abundant are lherzolites and dunites). The rest of springs are localized in volcanic and metamorphic rocks in the Kopaonik Mts. area. Although is the almost whole territory of the municipality Novo Brdo built by igneous rocks, cataclased gneiss-granite and serpentinized peridotites, the natural water emerges within the less abundant rock types: quartzite, marble, biotite gneisses and sericite–chlorite schists^20^.

## Materials and methods

### Sampling and analysis

Sampling water at 15 measuring sites was done in spring (April and May 2019) and autumn (September and October 2019). Preceded by tracking seasonal parameters, in order to avoid large variations in the concentration of the investigated elements, sampling was conducted once per month. At the very beginning of the study, the water temperature and pH value were measured following the recommendations rules from Policy book of the Republic of Serbia^21^. Temperature of water was measured with digital thermometer (Testo Se & KGaA, Germany), while pH value was measured by using a pH-meter (Microcomputer pH-vision 6071, JENCO Electronics. Ltd., Taiwan) with the combined electrode of type HI 1131 (Hanna Instruments).

Water was sampled in 1.5 L plastic bottle, two from each site following the sampling procedure provided^21,22^. One bottle was meant for chemical analysis, while the second was intended for radiological analysis. The bottles were closed and marked the exact time, date and place of sampling. For chemical analysis plastic bottles previously washed in a solution of 5% nitric acid (70 wt.% Fisher Scientific), followed by repeated rinsing with ultra pure Milli-Q water of > 15 MΩ/cm resistivity. Water samples for this analysis were stabilized by the addition of 1.5 mL HNO_3_ (70 wt.% Fisher Scientific) and stored in the refrigerator to analyzing samples on heavy metals^21^. For radological analysis bottles were filled by a thin jet of water up to the top, by which stirring of liquid should be avoided in the bottle, release of radon from the water and its accumulation in the free space under the cap, so they were closed immediately. Also, it is necessary that the time elapsed from bottling to measuring to be as short as it possible^22^.

### Detection of heavy metals

Analysis of heavy metals in water was conducted at the Faculty of Chemistry, University of Belgrade. The applied reagents were of the analytical purity (Merck, Darmstadt, Germany). Stock solutions of 1,000 mg/L concentration were prepared. Working solutions were prepared from stock solutions immediately before the analyses. For the preparation of standard solutions was used ultra pure Milli-Q water of > 15 MΩ/cm resistivity. The laboratory glassware and polyethylene containers were washed with the tap water before use, and then soaked in 6 M HNO_3_ solution few hours (or during the night) and rinsed several times with ultra pure water^23^.

The element´s contents were determined on a Thermo Scientific iCAP 6500 Duo ICP (Thermo Fisher Scientific, Cambridge, UK) as described previously by Kostic et al.^24^. To prepare calibration solutions for inductively coupled plasma optical emission spectrometry (ICP-OES) were used two plasma standard solutions. Multi-Element Plasma Standard Solution 4, Specpure, 1,000 µg/mL (Alfa Aesar GmbH & Co KG, Germany) and SS-Low Level Elements ICV Stock, ILM 05.2 ICS Stock 1 (VHG Labs, Inc-Part of LGC Standards, Manchester, NH 03103 USA). The analytical process quality control performed by EPA Methods 200.7 and LPC Solution Certified Reference Material (ULTRA Scientific, USA)^23^. The resulting concentrations were within 97–102%.

### Detection of radon

Detection of radon was conducted at the Laboratory for Testing Radioactivity of Samples and Doses of Ionizing and Non-Ionizing Radiation, at the Faculty of Sciences, University of Novi Sad. Concentration of radon in water samples was measured by the system RAD7 RAD H_2_O^25^, with lower limit of detection (LLD) less than 0.37 Bq/L, and minimal detectable activity (MDA) at 0.1 Bq/L^22^. It is possible to achieve this low activity value of background sample if completely eliminate all radon and its progeny before measurement and relative humidity during measurement was less than 8%. The inside of the RAD7 device consists of a semi-sphere of 0.7 L volume, which has a silicon implanted flat alpha-detector. A strong electric field inside of the voltage of range 2000—2,500 V directs the charged particles towards the surface of the detector. Their presence is reflected in the form of electric signals, whose intensity is proportional to the energies of detected alpha-particles. The RAD H_2_O method employs closed loop aeration scheme whereby the air volume and water volume are constant and independent of the flow rate. The air is recirculated through the water and continuously extracts the radon until a state of equilibrium develops. The RAD H_2_O system reaches this of equilibrium within about 5 min after which no more radon can be extracted from the water. The exact value of radon separation is almost always higher than 90%. Before every measuring the detector must be freed from the remaining radon, and also dry, which is achieved by blowing the air through the instrument^25^.

### Statistical analysis

Statistical analysis of results were done in the program packages Excel 2007 (Microsoft Office) and Statistica 8. Descriptive statistic and graph representation of results were done by using Excel. Pearson's correlation coefficient for the examined elements, as well as the multivariate cluster analysis of the springs with dendrogram, were determined in Statistica 8. The spatial distribution of heavy metals was mapped using Surfer 12.

## Results and discussions

### Heavy metals.

In Table 1 and Supplementary Table 1A (Appendix) the measuring results of the concentration (μg/L) of heavy metals As, Cd, Cr, Cu, Fe, Mn, Ni, Pb, Zn are shown. Actually, Table 1 represents minimum, maximum and mean values per municipality, of all measured parameters, and Supplementary Table 1A represents the average value of the triplicate measurement with relative standard deviations (RSD%) for each element form each sample. Supplementary Table 1A also presents their minimal, maximal and mean values for all samples. Negative values mean that these elements are practically non-existent (the concentration is less than 0.01 μg/L so they were not even taken into consideration, and they were treated as zero). The calculated average values differ depending on the researched measuring site of sampling.

**Table 1:** Minimum, maximum and mean values of measured parameters and calculated quantities, per municipalities.

	Zvečan			Leposavić			Novo brdo		
	Min	Max	Av	Min	Max	Av (with 0)	Min	Max	Av
As (μg/L)	4.11E − 01	1.451	1.012	− 9.755E − 01 (0)	33.74	6.5793	11135E − 01	8.596	3.3578
Cd (μg/L)	4.372E – 01	4.93E − 01	4.699E − 01	8.48E − 02	8.984E − 01	4.781E − 01	6.4E − 03	4.879E − 01	1.063E − 01
Cr (μg/L)	1.96E − 02	11.48	4.4949	1.954E − 01	12.47	3.1464	7.529E − 01	6.887	4.8563
Cu (μg/L)	1.071	2.178	1.4547	4.44E − 01	17.14	3.8142	− 5.85E − 01(0)	9.411E − 01	4.878E − 01
Fe (μg/L)	8.895E − 01	7.21	3.1658	5.686E − 01	255.9	55.0023	4.028E − 01	50.16	9.8284
Mn (μg/L)	4.688E − 01	5.562E − 01	5.040E − 01	3.901E − 01	4.237	1.6272	8.542E − 01	3.919	1.7669
Ni (μg/L)	7.274E − 01	6.605	2.7272	2.793E − 01	38.19	7.1731	1.332E − 01	8.167E − 01	4.133E − 01
Pb (μg/L)	1.999	41.72	25.513	2.906E − 01	8.781	2.2145	− 1.61 (0)	6.65	1.1305
Zn (μg/L)	1.684	13.76	5.886	− 1.286 (0)	6.006	1.955	3.1E − 03	122.3	21.434
U_ORAL_(As) (mg/kg/day)	1.09E − 05	3.84E − 05	2.68E − 05	0	8.92E − 04	1.74E − 04	3E − 06	2.27E − 04	8.87E − 05
U_ORAL_ (Cd) (mg/kg/day)	1.16E − 05	1.3E − 05	1.24E − 05	2.24E − 06	2.38E − 05	1.27E − 05	1.69E − 07	1.29E − 05	2.81E − 06
U_ORAL_ (Cr) (mg/kg/day)	5.18E − 07	3.04E − 04	1.19E − 04	5.17E − 06	3.3E − 04	8.32E − 05	1.99E − 05	1.82E − 04	1.28E − 04
U_ORAL_ (Cu) (mg/kg/day)	2.83E − 05	5.76E − 05	3.85E − 05	1.17E − 05	4.53E − 04	1.01E − 04	0	2.49E − 05	1.29E − 05
U_ORAL_ (Fe) (mg/kg/day)	2.35E − 05	1.91E − 04	8.38E − 05	1.5E − 05	6.765E − 03	1.454E − 03	1.06E − 05	1.326E − 03	2.6E − 04
U_ORAL_ (Mn) (mg/kg/day)	1.24E − 05	1.47E − 05	1.33E − 05	1.03E − 05	4.449E − 01	7.42E − 02	2.26E − 05	1.04E − 04	4.68E − 05
U_ORAL_ (Ni) (mg/kg/day)	1.92E − 05	1.75E − 04	7.22E − 05	7.38E − 06	1.01E − 03	1.9E − 04	3.52E − 06	2.16E − 05	1.09E − 05
U_ORAL_ (Pb) (mg/kg/day)	7.68E − 06	5.28E − 05	2.84E − 05	2.19E − 05	1.103E − 03	3.82E − 04	0	1.76E − 04	2.99E − 05
U_ORAL_ (Zn) (mg/kg/day)	4.45E − 05	3.64E − 04	1.56E − 04	0	1.59E − 04	5.17E − 05	8.2E − 08	3.233E − 03	5.67E − 04
NKR_ORAL_ (As)	3.62E − 02	1.279E − 01	8.9E − 02	0	2.9733	5.80E − 01	1E − 02	7.5752E − 01	2.959E − 01
NKR_ORAL_ (Cd)	2.31E − 02	2.61E − 02	2.48E − 02	4.484E − 03	4.7503E − 02	2.5281E − 02	3.38E − 04	2.5798E − 02	5.619E − 03
NKR_ORAL_ (Cr)	1.7E − 04	1.0117E − 01	3.961E − 02	1.722E − 03	1.0989E − 01	2.7728E − 02	6.635E − 03	6.0607E − 02	4.2797E − 02
NKR_ORAL_ (Cu)	5.66E − 03	1.152E − 02	7.69E − 03	2.348E − 03	9.0628E − 02	2.0168E − 02	0	4.976E − 03	2.579E − 03
NKR_ORAL_ (Fe)	3.36E − 05	2.72E − 04	1.19E − 04	2.15E − 05	9.665E − 03	2.077E − 03	1.52E − 05	1.894E − 03	3.71E − 04
NKR_ORAL_ (Mn)	8.85E − 05	1.05E − 04	9.51E − 05	1.18E − 05	8E − 04	2.95E − 04	1.61E − 04	7.4E − 04	3.34E − 04
NKR_ORAL_ (Ni)	9.62E − 04	8.731E − 03	3.605E − 03	3.69E − 04	5.0482E − 02	9.482E − 03	1.76E − 04	1.08E − 03	5.47E − 04
NKR_ORAL_ (Pb)	2.195E − 03	1.51E − 02	8.126E − 03	6.251E − 03	3.1514E − 01	1.0902E − 01	0	5.0231E − 02	8.539E − 03
NKR_ORAL_ (Zn)	1.48E − 04	1.213E − 03	5.19E − 04	0	5.29E − 04	1.72E − 04	2.73E − 07	1.0778E − 02	1.889E − 03
KR_ORAL_ (As)	1.63E − 05	5.75E − 05	4.01E − 05	0	1.338E − 03	2.61E − 04	4.5E − 06	3.41E − 04	1.33E − 04
KR_ORAL_ (Pb)	6.53E − 08	4.49E − 07	2.42E − 07	1.86E − 07	9.38E − 06	3.24E-6	0	1.49E − 06	2.53E − 07
KR_ORAL_ (Cr)	2.12E − 08	1.24E − 05	4.86E − 06	2.12E − 07	1.35E − 05	3.41E − 06	8.16E − 07	7.47–06	5.26E − 06
KR_ORAL_ (Cd)	7.05E − 08	7.95E − 08	7.58E − 08	1.37E − 08	1.45E − 07	7.71E − 08	1.03E − 09	7.87E − 08	1.71E − 08
KR_ORAL_ (Ni)	1.47E − 07	1.89E − 08	1.62E − 08	6.2E − 09	8.48E − 07	1.59E − 07	2.96E − 09	1.81E − 08	9.17E − 09
T (°C)	14	16	15	15	17	15.83	11	16	13.25
Ccorr (Bq/L)	27.6	46	35.53	0.34	30	11.5583	2.3	341	61.8
Eing (μS/year)	190	340	256.67	2	220	83	16	2,500	452.5
Einh (μS/year)	77.2	129.6	99.533	0.9	83.9	32.33	6.4	954	172.35

Based on these results, it is concluded that the values of analysed contents of heavy metals in some cases exceed the values given by the EU Directive^26^ and related to the Policy book of the Republic of Serbia^21,27^ and to the Policy about the quality and other requests for natural, mineral and table water^28^. In the Municipality of Zvečan the value of the concentration of Pb amounts to 32.82 μg/L (sample 2) and 41.72 μg/L (sample 3), bigger than the maximally allowed concentrations (MAC) suggested by the EU (10 μg/L)^26^, the Republic of Serbia (10 μg/L)^21,27,28^ and WHO 2006 (10 μg/L)^29^. The increased content of lead is probably the consequence of the vicinity of the flotation tailing ponds of MMCC “Trepča” which contains the increased concentration of sulphide minerals of lead^30^. In the municipality of Leposavić, the value of concentration of As amounts to 33.74 μg/L (sample 6). The value of Fe is 255 μg/L (sample 7), while the value of concentration of Ni is 38.19 μg/L (sample 6). This content can be expected, as a consequence of the vicinity of the mine whose beddings contain a significant part of primary oxide minerals Fe, Pb and As and secondary minerals with a certain part of presence, such as Ni, which is in accordance with data presented in literature^30–32^. The values of the other examined elements in the municipalities of Zvečan and Leposavić were lower than the prescribed values. In the municipality of Novo Brdo the concentration of examined metals was within the prescribed values, which can be explained by the bigger distance from the tailing pond and the source of pollution. During the sampling, pH values were measured, ranging from 6.6 to 7.6, while mean value was 7.04.

### Potential carcinogenic and non-carcinogenic risk assessment

Intake of toxic elements, especially their high concentration levels in drinking water, can have a harmful effect on human health.

Risk assessment, which components are the hazard and exposure, is the proceeding of evaluating the probability of any probable adverse health effects occurrence over a defined period of time. The health risk assessment of each potentially toxic metal is based on the quantification of the risk and is defined as carcinogenic (KR) or non- carcinogenic health risk (NKR). Two main toxicity risk factors for risk estimating are the slope factor (SF) for carcinogen risk and the reference dose (RfD) for non-carcinogen risk^33^. A slope factor SF is an upper bound on the increased cancer risk from a lifetime exposure to an agent by ingestion. Reference dose (RfD) is an estimate of a daily oral exposure to the human population that is likely to be without an appreciable risk of deleterious effects during a lifetime.

Risk characterization is the final step of health risk assessment, after hazard identification, hazard characterization, and exposure assessment. The health risk from groundwater consumption was assessed in relation to its non-carcinogenic as well as carcinogenic effects.

Potential non-carcinogenic risk from oral consumption (NKR_ORAL_) assessment can be determined using the following formula^34^:

1 $$ {\text{NKR}}_{{{\text{ORAL}}}} = { }\frac{{{\text{U}}_{{{\text{ORAL}}}} }}{{{\text{R}}_{{\text{f}}} {\text{D}}}},\,\, {\text{where is}}\,\, {\text{U}}_{{{\text{ORAL}}}} = { }\frac{{{\text{PPV }} \cdot {\text{c }}}}{{\text{PTM }}} $$

where PPV is the average water consumption per capita, 2 L daily; c is the concentration of elements included in the study (mg/L); PTM is the average weight of an adult consumer – 75.65 kg (based on available data from relevant medical institutions in Leposavić, Zvečan and Novo Brdo); RfD is the reference value for the intake of toxic elements (mg/kg/day), recommended by USEPA^34^; period of consumption is estimated at 365 days a year and exposure at 30 years; average exposure period is 10,950 days.

The carcinogenic risk from oral consumption (KR_ORAL_) for As, Pb, Cr, Cd and Ni (because of their potential long-term impact on human health) was calculated as the product of UORAL (mg/kg-day) times the SF (mg/kg/day)^−1^.

RfD and SF values for heavy metals are presented in Table 2.

**Table 2:** RfD and SF values for analysed heavy metals.

	RfD (mg/kg-day)	SF ((mg/kg/day)^−1^)
As	3 × 10^–4^	1.5
Cd	0.5 × 10^–3^	6.1 × 10^–3^
Cr	3 × 10^–3^	41 × 10^–3^
Cu	5 × 10^–3^	–
Fe	7 × 10^–1^	–
Mn	1.4 × 10^–1^	–
Ni	2 × 10^–2^	0.84 × 10^–3^
Pb	3.5 × 10^–3^	8.5 × 10^–3^
Zn	3 × 10^–1^	–

An acceptable level (that can be tolerated) of NKR_ORAL_ is ≤ 1, and of KR_ORAL_ is 1 × 10^–4^–1 × 10^–6^^33^

Arsenic has both carcinogenic and non-carcinogenic human health effect, so it is analysed from both points of view.

Results of U_ORAL_, NKR_ORAL_ and KR_ORAL_ are given in Tables 1 (min, max and mean values per municipalities), Supplementary Table 2A (Appendix), Supplementary Table 3A (Appendix) and Supplementary Table 4A (Appendix), respectively.

U_ORAL_ values range from 0 to 0.00677 mg/kg/day. Based on these values, NKR_ORAL_ and KR_ORAL_ were calculated at all 15 measuring sites.

NKR_ORAL_ values range from 0 to 2.973. The maximum value (2.973) refers to As at sample 6 and it can be confirmed that this element poses a significant carcinogenic risk.

The values for KR_ORAL_ give a completely different picture. For As, it ranges from 0 to 0.0013. Arsenic poses a significant carcinogenic risk to the health of residents. The most threatened sample is 6, followed by 10, 14 and 13, marked in bold (Supplementary Table 4A). Values of parameters with acceptable risk are marked in italic bold (Supplementary Table 4A).

### Radon

Since the concentration of radon in water could not be measured at the sampling site in Table 1 shows corrected value of radon concentration Ccorr determined by the formula^22^:

2 $$ {\text{C}}_{{{\text{corr}}}} = {\text{ C}}_{{\text{o}}} \cdot\delta , $$

where δ = e^λt^, radon decay λ = 0.00756 h^−1^ and C_o_ (Bq/L) value was measured in the laboratory after several days and t defines time elapsed from sampling to the laboratory analysis. Water temperature, effective dose from ingestion E_ing_ and effective dose from inhalation E_inh_ are also the parameters presented in Table 1 (min, max and mean values per municipalities) and Supplementary Table 5A.

Carcinogenic effect of radon, in the long-term sense, refers to determining the total effective dose of internal radon radiation dissolved in water, which consists of two components: the first one being defined by the effective dose from ingestion, whereas the second one being defined by the effective dose from inhalation. Radon-rich water goes directly to the stomach, wherefrom radon could penetrate through the stomach walls into the body. One part of the radionuclides could stay at the same place for a long time, while others could bind to macrophages and then be transferred to lymph cells. These cells can receive radiation doses of alpha particles emitted by the decay of radon and its short-lived progeny in the stomach walls^35^. Effective dose from ingestion of radon and its progeny relates to gastric tissue, expressed in mSv/y is determined in the following way:

3 $$ {\text{E}}_{{{\text{ing}}}} = {\text{ K }} \times {\text{ C}}_{{{\text{Rn}}}} \times {\text{ KM }} \times {\text{ t}} $$

where K is the conversion factor of 10–8 Sv/Bq for adults; CRn is radon concentration in water (Bq/L); KM is the consumption factor (optimally 2 L/day) ^36^ and t period of 365 days^37^.

It is known that radon leaves the water very quickly and thus increases its presence in indoor air^22^. Since the spring water is used by the population for drinking and other household purposes, the effective dose of inhalation was determined as followed: 1 Bq/m^3^ radon in air^38^, with equilibrium factor 0.4 and occupation factor of 0.8 gives an effective dose to the lung of 25 µSv/y. Assuming that the ratio of radon concentration released from water into air and a radon concentration in water equal to 10^–4^, the conversion factor from unit concentration from radon et equilibrium is 2.8 µSv/Bq m^3^^38^.

The results indicate that in the sampled spring waters radon concentration ranges from 0.34 ± 0.12 Bq/L (sample 8) to 341 ± 35 Bq/L (sample 11), with a mean value of 36.44 Bq/L, which is below the reference level of 100 Bq/L^37,39^. This somewhat higher radon concentration is a consequence of the geological structure of the Zvečan municipality’s terrain; the structure of the northeastern terrain is dominant in Paleozoic shales (samples 2 and 3) whereas the western part of the terrain is characterized by igneous rocks (sample 1)^20^. Radon concentration in water sampled at sites 4, 5 and 6 in the municipality of Leposavić is higher than samples from sites 7, 8, and 9. That is a result of the geological structure of the terrain itself, where Neogene sediments and magmatic rocks prevail^20^. In the area of the municipality of Novo Brdo, exceptionally high radon concentration in the water 341 ± 35 Bq/L (sample 11) was measured at only one site, while at others radon levels were low. This high radon concentration is a consequence of the geological structure of the terrain where the spring is located. Although the total area of the municipality of Novo Brdo is abundant with magmatic rocks, granite and marble, the terrain’s geological structure at the actual source is dominant with quartz, marble and granitic rocks of different metamorphic origin^40^. If the mean value of radon concentration presented in this paper is compared with the data from literature^41–45^ it can be seen to be slightly higher. This can be justified by the geological structure of the terrain.

Temperature ranges from 11 ℃ (sample 12) to 17 ℃ (sample 6), with a mean value of 14.6 ℃, which puts them into the group of cold ground waters^22^.

The value of effective dose from ingestion ranges from 2 μSv/y (sample 8) to 2,500 μSv/y (sample 11), with a mean value of 260 μSv/y, which is above the recommended value of 100 μSv/y^40^. It could be argued that, from a radiological aspect, the water from all springs, except for the one from the village of Vlasce in the municipality of Novo Brdo (sample 11), is wholesome and can be used for drinking as well as other purposes. Regarding the radiation dose from radon inhalation, it mainly derives from the short-lived radon progeny settled in bronchi since most radon is expelled by exhaling. The effective dose from inhalation ranges from the minimal value of 0.9 μSv/y (sample 8) to 954 μSv/y (sample 11), with a mean value of 101.8 μSv/y.

### Statistical analyses

The correlation of measured parameters: heavy metals, radon in water and pH level in water defines the value of Pearson’s correlation coefficient—r, which is presented in Table 3.

**Table 3:** Pearson’s correlation coefficient value (r) of the measured parameters.

	As	Cd	Cr	Cu	Fe	Mn	Ni	Pb	Zn	Rn	pH
As	1.000	0.5 × 10^–4^	0.422	0.015	0.015	0.032	0.005	0.049	0.02	0.001	0.007
Cd		1.000	0.001	0.451	0.483	**0.640**	0.005	0.013	0.022	0.075	0.040
Cr			1.000	0.053	0.080	0.194	0.046	0.352	0.060	**0.827**	0.006
Cu				1.000	**0.948**	**0.642**	0.023	0.586	0.001	0.029	0.121
Fe					1.000	**0.769**	0.043	0.520	0.8 × 10^–4^	0.063	0.242
Mn						1.000	0.046	0.352	0.023	**0.827**	0.006
Ni							1.000	0.066	0.046	0.038	0.017
Pb								1.000	0.042	0.378	0.323
Zn									1.000	0.335	0.002
Rn										1.000	0.003

Significant values are in bold

The most evident is the correlation of iron and copper—0.948, then copper and manganese. There is also a positive correlation between manganese with cadmium—0.640 and iron—0.769. The values of correlation coefficient of lead with iron and copper of 0.520 and 0.586, respectively, are another indicator of their common origin. As regards the correlation between radon and heavy elements, the correlation between radon and chromium—0.827 and manganese 0.827 singles out. The data from Table 3 indicates that the pH values of the investigated samples do not have any significant impact on the measured values of the present elements of heavy metals and radon, although a certain correlation of pH values and lead—0.323 and iron—0.242 could be argued. Based on the small values of the correlation coefficients obtained for some of examined elements, it can be concluded that they are of natural origin.

**Figure 2:**
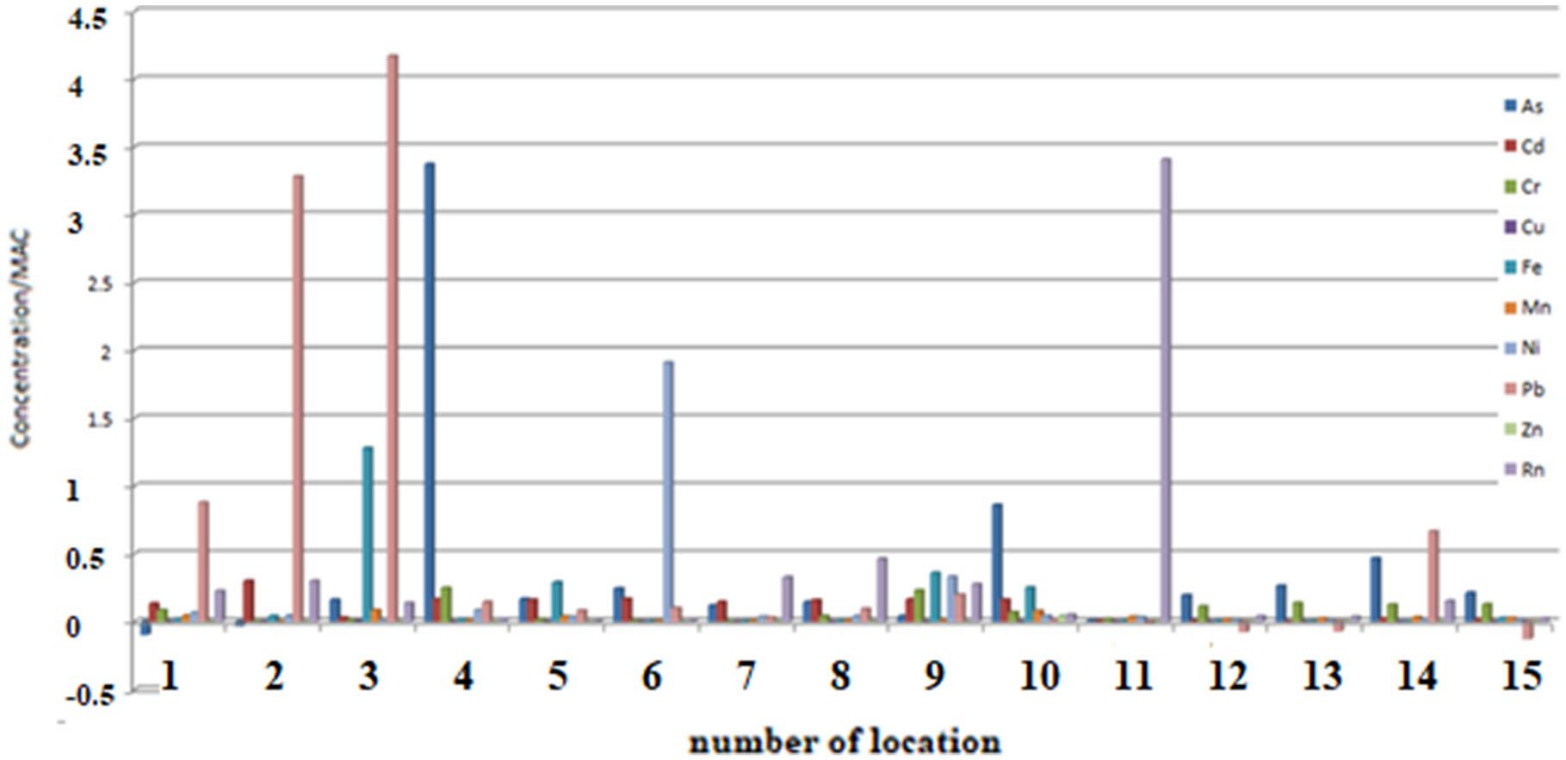
Concentrations of heavy metals and radon compared to MAC and in accordance with EU, WHO and the Republic of Serbia’s policies in sampled natural spring waters at 15 measuring sites.

Concentrations of the investigated elements of heavy metals and radon compared to MAC and in accordance with Policies^20,26,27,37^ at all measuring sites (1–15) at the investigated locations are shown in Fig. 2.

From the Fig. 2 it can be seen how many times each individual element increases concentration over the recommended value. Thus, Pb in sample 2 has 3.2 times higher concentracion and sample 3 has four times higher concentracion than the recommended value of 10 μg/L. In sample 4 Ni is almost two times higher than the recommended value of 20 μg/L. In sample 6 As stands out in enhanced concentration, it has as much as 3.3 times higher concentration than the recommended − 10 μg/L. In sample 11, the pronounced concentration of Rn is 3.5 times higher than the recommended value of 100 Bq/L. Concentrations of other tested elements at all 15 selected measuring sites do not deviate significantly from the recommended one, or are found in traces, so their relationships should not be commented.

**Figure 3:**
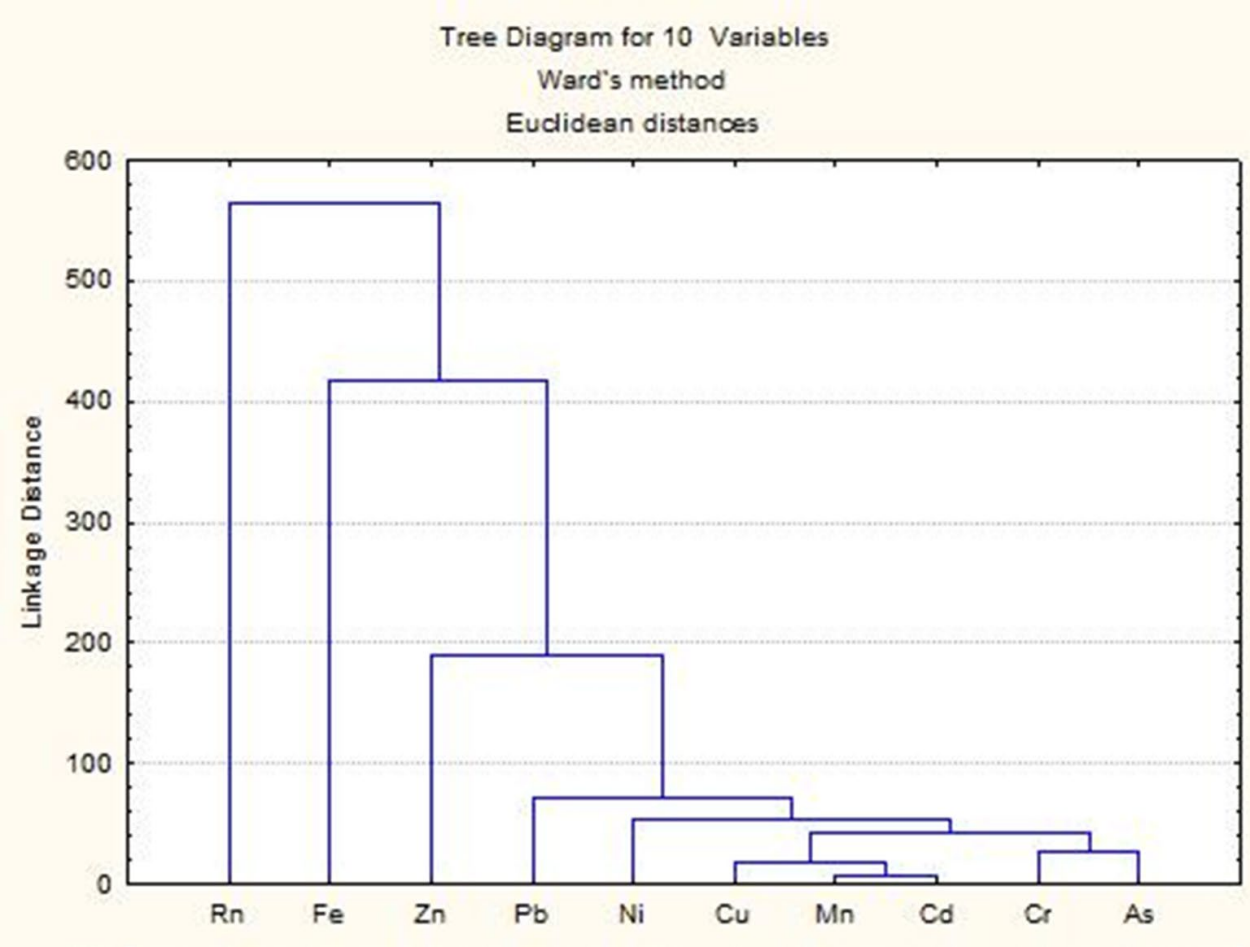
Dendrogram of heavy metals (StatSoft Statistica 8 https://www.statsoft.de/en/statistica/statistica-software).

Figure 3 shows the results of the multivariate cluster analysis as a dendrogram. According to the results, the primary clusters of heavy metals As–Cr and Cu–Mn–Cd indicate the common origin of the minerals: the first from mining activities in vicinity of springs, and second from inustry, both indicate anthropogenic impact.

**Figure 4:**
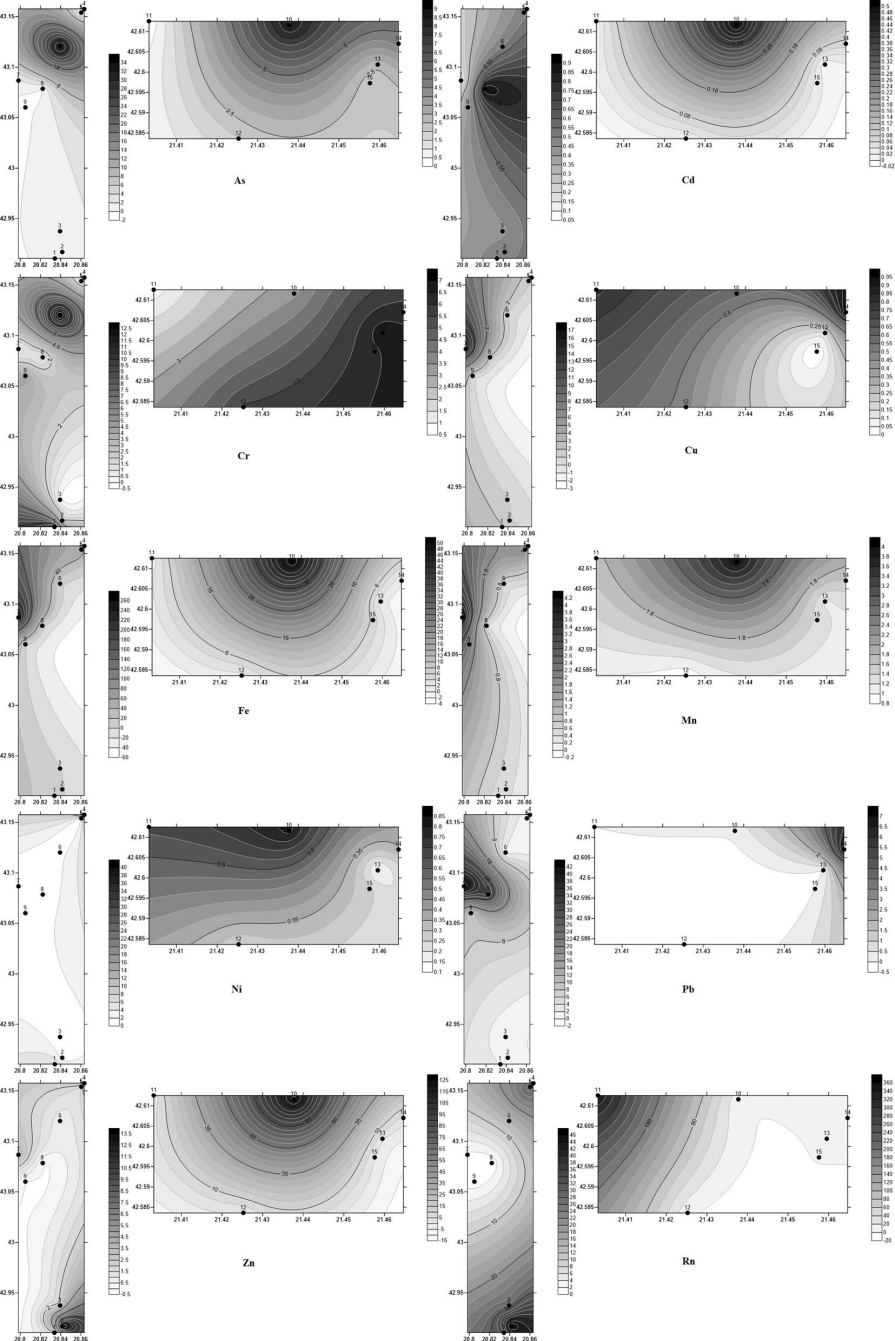
Spatial distribution of heavy metals and radon (heavy metals in μg/L and Rn in Bq/L) (Golden Software Surfer12 https://www.goldensoftware.com/products/surfer).

Figure 4 shows the spatial distribution of each heavy metal and Rn.

Principal component analysis (PCA), mathematical tool used to reduce the number of variables while retaining the original variability of the data, was performed to explain the cause, that is, to define the dominant factor affecting the measured concentrations of heavy metals in the water samples at all measuring sites. It can be noticed, already on the basis of correlations, and then from PCA results, that at all measuring points Cu, Fe, Pb and Mn are consequences of the same pollution factor (mining), while Cr and As are the consequence of some other source of pollution (industry). Other four elements are consequences of other four different factors (Table 4). Negative values occur in variables that are inversely related.

**Table 4:** Results of PCA analyses of heavy metals in the water samples.

	F1	F2	F3	F4	F5	F6	F7	F8	F9	F10
As	0.248689	**0.762997**	− 0.116944	− 0.104675	− 0.302493	− 0.414501	0.251683	0.068414	− 0.003393	− 0.002817
Cd	0.147736	0.014845	− **0.816690**	0.291957	0.262480	− 0.347123	− 0.101032	− 0.132256	0.092755	− 0.006250
Cr	0.436978	**0.711069**	0.108911	− 0.381773	− 0.083992	0.087257	− 0.359959	− 0.027694	0.027760	0.006493
Cu	− **0.917217**	0.038311	− 0.094467	− 0.270910	− 0.197190	− 0.060088	− 0.051314	− 0.126009	− 0.101177	− 0.060711
Fe	− **0.957039**	0.131448	− 0.023132	− 0.083394	− 0.160759	− 0.014137	0.035250	− 0.166459	0.003412	0.065592
Mn	− **0.786385**	0.262207	0.312067	0.386607	− 0.064117	0.072343	− 0.020877	0.067114	0.226424	− 0.021069
Ni	0.192399	− 0.363365	− 0.509882	0.134457	− **0.717535**	0.160503	− 0.082175	0.067285	0.031216	0.004336
Pb	− **0.800678**	− 0.045183	− 0.355249	− 0.268953	0.196682	− 0.174604	− 0.121214	0.269176	− 0.042559	0.014337
Zn	− 0.140054	0.386261	0.049370	**0.887726**	− 0.019438	0.031647	− 0.104602	0.028535	− 0.165946	0.004476
Rn	0.102074	− 0.467276	0.581351	0.083932	− 0.187886	− **0.605144**	− 0.156858	− 0.008894	0.002036	0.005675

Significant values are in bold

## Conclusion

This paper presents the results of research conducted at different locations and immediate vicinity of mining and industrial activities of northern and southeastern part of Kosovo. The results of measuring the concentration of heavy metals As, Cd, Cr, Cu, Fe, Mn, Ni, Pb, Zn, radon, pH value and temperature are estimated in the samples of spring waters collected in the municipalities of Zvečan, Leposavić and Novo Brdo. The analyzed samples show a high concentration level of metals As, Pb, Fe and Ni, which indicates the pollution of the environment, as a consequence of the vicinity of the mining areas and industrial activities to the investigated areas. It is well-known that intake of heavy metals, especially those with high concentration levels present in drinking water, can have a detrimental effect on people’s health. However, the value of the calculated coefficients of potential non-carcinogenic risk is mainly below 1 (except one locality for As), and the values of the calculated coefficients of potential carcinogenic risk for four locations are above 1 × 10^–4^, indicating that these waters can not be used for drinking and other purposes in households.

Radon concentration ranges from 0.34 ± 0.12 Bq/L (sample 8) to 341 ± 35 Bq/L (sample 11), with a mean value of 36.44 Bq/L, which is, compared to MAC of 100 Bq/L recommended by the WHO, below the reference level. The exceptionally high levels of radon in water of 341 ± 35 Bq/L in the municipality of Novo Brdo (sample 11) is a consequence of the geological structure of the terrain where the spring is located. The value of the effective dose from ingestion ranges from 2.0 μSv/year (sample 8) to 2,500 μSv/year (sample 11), with a mean value of 260 μSv/year, which is above the recommended value of 100 μSv/year^46^. The results of these studies are the first to give an image of spring water quality and are the first to measure radon in water in this area, so they can serve as a starting point for the formation of a radon map from Kosovo.

Although water is radiological correct, authors conclude, considering chemical analysis, that water from investigated sources is not recommended for wider use. The presence of heavy metals and radon requires their constant monitoring and control, strengthening ecological consciousness about the real risk from high concentrations of toxic elements on people’s health and introducing environmentally friendly production.

## Supplementary information

Supplementary information
