# Supplementary material for: Heavy metals and radon content in spring water of Kosovo
Source: Sci Rep. 2020 Jun 25;10:10359. doi: 10.1038/s41598-020-67371-1 (PMC7316983; doi:10.1038/s41598-020-67371-1)
Supplement: Supplementary file 1 — Supplementary information [file 41598_2020_67371_MOESM1_ESM.pdf]

**Heavy metals and radon content in spring water of Kosovo**

*Smiljana Marković, Biljana Vučković, Ljiljana Nikolić-Bujanović, Sanja Mrazovac Kurilić, Nataša Todorovic, Jovana Nikolov, Anja Jokić, Boban Đokić*

**Table 1A** Municipalities and measuring sites: **Zvečan**: 1. Zvečan, Gvozdićka česma; 2. Rudare, Sokolica; 3. Grabovac; **Leposavić**: 4. Lokvice; 5. Koporiće; 6. Kijevčiće; 7. Popovce; 8. Ravnište; 9. Jevtiće; **Novo Brdo**: 10. Nova Kolonija; 11. Vlasce; 12. Trnićevce; 13. Vlajkovci; 14. Utmanovci; 15. Reka.

| No of sample | | As (µg/L) | Cd (µg/L) | Cr (µg/L) | Cu (µg/L) | Fe (µg/L) | Mn (µg/L) | Ni (µg/L) | Pb (µg/L) | Zn (µg/L) |
|---|---|---|---|---|---|---|---|---|---|---|
| **Zvečan** | | | | | | | | | | |
| 1 | **Concentration** | **0.4110** | **0.4930** | **11.48** | **2.178** | **7.210** | **0.4869** | **6.605** | **1.999** | **1.684** |
| | %RSD | 187.1 | 2.178 | 1.660 | 9.120 | 20.03 | 8.411 | 0.9185 | 20.84 | 1.065 |
| 2 | **Concentration** | **1.451** | **0.4795** | **1.985** | **1.115** | **1.398** | **0.4688** | **0.8491** | **32.82** | **13.76** |
| | %RSD | 110.4 | 8.583 | 9.999 | 7.349 | 70.18 | 7.730 | 11.37 | 61.19 | 2.792 |
| 3 | **Concentration** | **1.174** | **0.4372** | **0.0196** | **1.071** | **0.8895** | **0.5562** | **0.7274** | **41.72** | **2.214** |
| | %RSD | 50.96 | 14.04 | 201.70 | 23 .88 | 42.12 | 11 .32 | 31 .94 | 293 .1 | 3.468 |
| **Leposavić** | | | | | | | | | | |
| 4 | **Concentration** | **2.459** | **0.5055** | **0.1954** | **1.305** | **0.5686** | **0.3901** | **38.19** | **1.003** | **6.006** |
| | %RSD | 28.88 | 1.651 | 181.0 | 3.798 | 27.07 | 6.334 | 0.7048 | 40.07 | 1.102 |
| 5 | **Concentration** | **1.669** | **0.4815** | **0.7282** | **1.280** | **57.99** | **1.914** | **0.6804** | **0.8275** | **1.397** |
| | %RSD | 14.42 | 2.846 | 30.94 | 24.02 | 2.139 | 1.277 | 14.01 | 31.82 | 1.113 |
| 6 | **Concentration** | **33.74** | **0.4987** | **12.47** | **0.9094** | **3.465** | **0.5222** | **1.688** | **1.447** | **0.7431** |
| | %RSD | 2.967 | 8.509 | 2.103 | 29.64 | 99.50 | 13.92 | 1.189 | 32.98 | 1.774 |
| 7 | **Concentration** | **1.608** | **0.0848** | **0.5375** | **17.14** | **255.9** | **4.237** | **0.2793** | **0.2906** | **3.581** |
| | % RSD | 52.94 | 18.26 | 48.32 | 2.829 | 0.4389 | 0.9651 | 110.3 | 1.474 | 1.474 |
| 8 | **Concentration** | **-0.3224** | **0.8984** | **0.6582** | **1.807** | **8.376** | **0.4449** | **0.8568** | **0.9376** | **-0.027** |
| | % RSD | 491.1 | 113.2 | 49.37 | 66.25 | 3.906 | 27.03 | 102.6 | 6.726 | 2.914 |
| 9 | **Concentration** | **-0.9755** | **0.3999** | **4.289** | **0.4440** | **3.714** | **2.255** | **1.344** | **8.781** | **-1.286** |
| | % RSD | 90.85 | 85.64 | 8.081 | 97.13 | 9.714 | 1.887 | 28.18 | 18.42 | 20.26 |
| **Novo Brdo** | | | | | | | | | | |
| 10 | **Concentration** | **8.596** | **0.4879** | **3.384** | **0.5673** | **50.16** | **3.919** | **0.8167** | **0.1330** | **122.3** |
| | % RSD | 13.44 | 2.804 | 75.72 | 38.26 | 3.617 | 9.220 | 27.62 | 403.1 | 0.1866 |
| 11 | **Concentration** | **0.1135** | **0.0194** | **0.7529** | **0.8143** | **0.9023** | **1.707** | **0.6636** | **-1.610** | **2.190** |
| | % RSD | 806.0 | 245.1 | 297.9 | 7.695 | 135.0 | 65.53 | 21.05 | 28.07 | 3.212 |
| 12 | **Concentration** | **1.965** | **0.0219** | **5.559** | **0.4707** | **1.294** | **0.8542** | **0.2599** | **-0.7592** | **0.4167** |
| | % RSD | 46.83 | 244.9 | 57.33 | 109.7 | 190.0 | 57.32 | 99.09 | 123.1 | 5.456 |
| 13 | **Concentration** | **2.650** | **0.0064** | **6.887** | **0.1333** | **0.7822** | **1.161** | **0.1332** | **-0.7318** | **0.5184** |
| | % RSD | 47.04 | 689.4 | 30.10 | 207.2 | 334.6 | 145.8 | 174.9 | 61.98 | 8.618 |

| | | | | | | | | | | |
|---|---|---|---|---|---|---|---|---|---|---|
| 14 | Concentration | **4.688** | **0.0703** | **6.194** | **0.9411** | **0.4028** | **1.595** | **0.3397** | **6.650** | **3.176** |
| | % RSD | 37.91 | 33.94 | 6.226 | 57.88 | 250.1 | 64.06 | 40.53 | 3.796 | 2.113 |
| 15 | Concentration | **2.134** | **0.0317** | **6.361** | **-0.5853** | **5.429** | **1.365** | **0.2668** | **-1.296** | **0.0031** |
| | % RSD | 11.55 | 115.8 | 28.70 | 33.50 | 33.02 | 125.2 | 52.40 | 13.56 | 1647 |
| **MAC**[21,28] | | 10 | 3 | 50 | 2000 | 200 | 50 | 20 | 10 | 3000 |
| **Min** | | -0.976 | 0.0064 | 0.0196 | -0.5850 | 0.4028 | 0.3901 | 0.1332 | -1.610 | -1.286 |
| **Mean** | | 4.093 | 0.3277 | 4.10 | 1.973 | 26.57 | 1.458 | 3.580 | 6.147 | 10.45 |
| **Max** | | 33.74 | 0.8984 | 12.47 | 17.14 | 255.9 | 4.237 | 38.19 | 41.72 | 122.3 |

**Table 2**A UORAL values for 9 heavy metals in in natural spring water from 15 measuring sites (mg/kg/day)

| Measuring sites | As | Cd | Cr | Cu | Fe | Mn | Ni | Pb | Zn |
|---|---|---|---|---|---|---|---|---|---|
| **Zvečan** | | | | | | | | | |
| **1** | 1.09E-05 | 1.3E-05 | 0.000304 | 5.76E-05 | 0.000191 | 1.29E-05 | 0.000175 | 5.28E-05 | 4.45E-05 |
| **2** | 3.84E-05 | 1.27E-05 | 5.25E-05 | 2.95E-05 | 3.7E-05 | 1.24E-05 | 2.24E-05 | 2.48E-05 | 0.000364 |
| **3** | 3.1E-05 | 1.16E-05 | 5.18E-07 | 2.83E-05 | 2.35E-05 | 1.47E-05 | 1.92E-05 | 7.68E-06 | 5.85E-05 |
| **Leposavić** | | | | | | | | | |
| **4** | 6.5E-05 | 1.34E-05 | 5.17E-06 | 3.45E-05 | 1.5E-05 | 1.03E-05 | 0.00101 | 2.65E-05 | 0.000159 |
| **5** | 4.41E-05 | 1.27E-05 | 1.93E-05 | 3.38E-05 | 0.001533 | 5.06E-05 | 1.8E-05 | 2.19E-05 | 3.69E-05 |
| **6** | 0.000892 | 1.32E-05 | 0.00033 | 2.4E-05 | 9.16E-05 | 1.38E-05 | 4.46E-05 | 3.83E-05 | 1.96E-05 |
| **7** | 4.25E-05 | 2.24E-06 | 1.42E-05 | 0.000453 | 0.006765 | 0.000112 | 7.38E-06 | 0.001103 | 9.47E-05 |
| **8** | 0 | 2.38E-05 | 1.74E-05 | 4.78E-05 | 0.000221 | 0.4449 | 2.27E-05 | 0.000868 | 0 |
| **9** | 0 | 1.06E-05 | 0.000113 | 1.17E-05 | 9.82E-05 | 5.96E-05 | 3.55E-05 | 0.000232 | 0 |
| **Novo Brdo** | | | | | | | | | |
| **10** | 0.000227 | 1.29E-05 | 8.95E-05 | 1.5E-05 | 0.001326 | 0.000104 | 2.16E-05 | 3.52E-06 | 0.003233 |
| **11** | 3E-06 | 5.13E-07 | 1.99E-05 | 2.15E-05 | 2.39E-05 | 4.51E-05 | 1.75E-05 | 0 | 5.79E-05 |
| **12** | 5.19E-05 | 5.79E-07 | 0.000147 | 1.24E-05 | 3.42E-05 | 2.26E-05 | 6.87E-06 | 0 | 1.1E-05 |
| **13** | 7.01E-05 | 1.69E-07 | 0.000182 | 3.52E-06 | 2.07E-05 | 3.07E-05 | 3.52E-06 | 0 | 1.37E-05 |
| **14** | 0.000124 | 1.86E-06 | 0.000164 | 2.49E-05 | 1.06E-05 | 4.22E-05 | 8.98E-06 | 0.000176 | 8.4E-05 |
| **15** | 5.64E-05 | 8.38E-07 | 0.000168 | 0 | 0.000144 | 3.61E-05 | 7.05E-06 | 0 | 8.2E-08 |

**Table 3**A NKRORAL values for 9 heavy metals in in natural spring water from 15 measuring sites (non-dimensional value)

| Measuring sites | As | Cd | Cr | Cu | Fe | Mn | Ni | Pb | Zn |
|---|---|---|---|---|---|---|---|---|---|
| **Zvečan** | | | | | | | | | |
| **1** | 0.036219 | 0.026067 | 0.101168 | 0.011516 | 0.000272 | 9.19E-05 | 0.008731 | 0.0151 | 0.000148 |
| **2** | 0.127869 | 0.025354 | 0.017493 | 0.005896 | 5.28E-05 | 8.85E-05 | 0.001122 | 0.007082 | 0.001213 |
| **3** | 0.103459 | 0.023117 | 0.000173 | 0.005663 | 3.36E-05 | 0.000105 | 0.000962 | 0.002195 | 0.000195 |
| **Leposavić** | | | | | | | | | |
| **4** | 0.216699 | 0.026728 | 0.001722 | 0.0069 | 2.15E-05 | 7.37E-05 | 0.050482 | 0.007576 | 0.000529 |
| **5** | 0.147081 | 0.025459 | 0.006417 | 0.006768 | 0.00219 | 0.000361 | 0.000899 | 0.006251 | 0.000123 |
| **6** | **2.973338** | 0.026369 | 0.109892 | 0.004808 | 0.000131 | 9.86E-05 | 0.002231 | 0.01093 | 6.55E-05 |
| **7** | 0.141705 | 0.004484 | 0.004737 | 0.090628 | 0.009665 | 0.0008 | 0.000369 | 0.315135 | 0.000316 |
| **8** | 0 | 0.047503 | 0.0058 | 0.009555 | 0.000316 | 1.18E-05 | 0.001133 | 0.247908 | 0 |
| **9** | 0 | 0.021145 | 0.037797 | 0.002348 | 0.00014 | 0.000426 | 0.001777 | 0.066328 | 0 |

| | | | | | | | | | |
|---|---|---|---|---|---|---|---|---|---|
| **Novo Brdo** | | | | | | | | | |
| **10** | 0.757523 | 0.025798 | 0.029822 | 0.003 | 0.001894 | 0.00074 | 0.00108 | 0.001005 | 0.010778 |
| **11** | 0.010002 | 0.001026 | 0.006635 | 0.004306 | 3.41E-05 | 0.000322 | 0.000877 | 0 | 0.000193 |
| **12** | 0.173166 | 0.001158 | 0.048989 | 0.002489 | 4.89E-05 | 0.000161 | 0.000344 | 0 | 3.67E-05 |
| **13** | 0.233531 | 0.000338 | 0.060692 | 0.000705 | 2.95E-05 | 0.000219 | 0.000176 | 0 | 4.57E-05 |
| **14** | 0.41313 | 0.003717 | 0.054585 | 0.004976 | 1.52E-05 | 0.000301 | 0.000449 | 0.050231 | 0.00028 |
| **15** | 0.188059 | 0.001676 | 0.056056 | 0 | 0.000205 | 0.000258 | 0.000353 | 0 | 2.73E-07 |

**Table 4A** KRORAL values for 5 heavy metals in spring in natural spring water from 15 measuring sites (non-dimensional value)

| **Measuring sites** | **As** | **Pb** | **Cr** | **Cd** | **Ni** |
|---|---|---|---|---|---|
| **Zvečan** | | | | | |
| **1** | ***1.63E-05*** | 4.49E-07 | ***1.24E-05*** | 7.95E-08 | 1.47E-07 |
| **2** | ***5.75E-05*** | 2.11E-07 | ***2.15E-06*** | 7.73E-08 | 1.89E-08 |
| **3** | ***4.66E-05*** | 6.53E-08 | 2.12E-08 | 7.05E-08 | 1.62E-08 |
| **Leposavić** | | | | | |
| **4** | ***9.75E-05*** | 2.25E-07 | 2.12E-07 | 8.15E-08 | 8.48E-07 |
| **5** | ***6.62E-05*** | 1.86E-07 | 7.89E-07 | 7.77E-08 | 1.51E-08 |
| **6** | **0.001338** | 3.25E-07 | ***1.35E-05*** | 8.04E-08 | 3.75E-08 |
| **7** | ***6.38E-05*** | ***9.38E-06*** | 5.83E-07 | 1.37E-08 | 6.2E-09 |
| **8** | 0 | ***7.38E-06*** | 7.13E-07 | 1.45E-07 | 1.9E-08 |
| **9** | 0 | ***1.97E-06*** | ***4.65E-06*** | 6.45E-08 | 2.98E-08 |
| **Novo Brdo** | | | | | |
| **10** | **0.000341** | 2.99E-08 | ***3.67E-06*** | 7.87E-08 | 1.81E-08 |
| **11** | ***4.5E-06*** | 0 | 8.16E-07 | 3.13E-09 | 1.47E-08 |
| **12** | ***7.79E-05*** | 0 | ***6.03E-06*** | 3.53E-09 | 5.77E-09 |
| **13** | **0.000105** | 0 | ***7.47E-06*** | 1.03E-09 | 2.96E-09 |
| **14** | **0.000186** | ***1.49E-06*** | ***6.71E-06*** | 1.13E-08 | 7.54E-09 |
| **15** | ***8.46E-05*** | 0 | ***6.89E-06*** | 5.11E-09 | 5.92E-09 |

**Table 5A** Summed-up radon Rn-222 measurement results in the samples of natural spring waters collected at the investigated measuring sites

| No of sample | T ($^{o}$C) | $C_{corr}$(Bq/l) | $E_{ing}$ (μSv/y) | $E_{inh}$ (μSv/y) |
|---|---|---|---|---|
| **Zvečan** | | | | |
| **1** | 15 | 33±3 | 240±24 | 91.8±8 |
| **2** | 16 | 46±5 | 340±30 | 129.6±14 |
| **3** | 14 | 27.6±2.8 | 190±20 | 77.2±7.8 |
| **Leposavić** | | | | |
| **4** | 16 | 22.7±2.3 | 160±16 | 63.6±6.4 |
| **5** | 15 | 30±3 | 220±22 | 83.9±8.4 |
| **6** | 17 | 13.8±1.4 | 100±10 | 38.7±3.9 |
| **7** | 16 | 1.6±0.5 | 10±3 | 4.4±1.4 |
| **8** | 15 | 0.34±0.12 | 2.0±0.7 | 0.9±0.3 |
| **9** | 16 | 0.91±0.15 | 6.0±1.0 | 2.5±0.4 |
| **Novo Brdo** | | | | |
| **10** | 16 | 5.1±0.5 | 37±3 | 11.1±1.4 |
| **11** | 11.5 | 341±35 | 2500±260 | 954±98 |
| **12** | 11 | 3.9±0.4 | 28.0±2.9 | 10.9±1.1 |
| **13** | 15.5 | 3.4±0.4 | 24.0±2.8 | 9.4±1.1 |
| **14** | 12 | 15.1±0.9 | 110±6 | 42.3±2.5 |
| **15** | 13.5 | 2.3±0.4 | 16.0±2.8 | 6.4±1.1 |
| **Min** | 11 | 0.34±0.12 | 2.0 | 0.9 |
| **Mean** | 14.6 | 36.44 | 260 | 101.8 |
| **Max** | 17 | 340.72±35 | 2500 | 954 |
